# Jigsaw Puzzling Taps Multiple Cognitive Abilities and Is a Potential Protective Factor for Cognitive Aging

**DOI:** 10.3389/fnagi.2018.00299

**Published:** 2018-10-01

**Authors:** Patrick Fissler, Olivia Caroline Küster, Daria Laptinskaya, Laura Sophia Loy, Christine A. F. von Arnim, Iris-Tatjana Kolassa

**Affiliations:** ^1^Institute of Psychology and Education, Clinical and Biological Psychology, Ulm University, Ulm, Germany; ^2^Department of Neurology, Ulm University, Ulm, Germany; ^3^Clinic for Neurogeriatrics and Neurological Rehabilitation, University and Rehabilitation Hospital Ulm, Ulm, Germany; ^4^Department of Media Psychology, University of Hohenheim, Stuttgart, Germany

**Keywords:** jigsaw puzzles, visuospatial cognition, cognitive aging, cognitive intervention, cognitive enrichment, dementia, neurocognitive disorders, cognitive impairment

## Abstract

Prevention of neurocognitive disorders is currently one of the greatest unmet medical challenges. The cognitive effects of solving jigsaw puzzles (JPs) have not been studied so far, despite its frequent use as a leisure activity in all age cohorts worldwide. This study aimed at closing this gap between a lack of science and a frequent real-world use by investigating the cognitive abilities recruited by JP as well as the cognitive benefits of lifetime and 30-day JP experience. A total of 100 cognitively healthy adults (≥50 years of age) were randomized to either a 30-day home-based JP intervention (≥1 h/day) plus four sessions of cognitive health counseling (JP group) or four sessions of cognitive health counseling only (counseling group). We measured global visuospatial cognition by averaging the scores of eight *z*-standardized visuospatial cognitive abilities (perception, constructional praxis, mental rotation, speed, flexibility, working memory, reasoning, and episodic memory). JP skill was assessed with an untrained 40 piece JP and lifetime JP experience with retrospective self-report. JP skill was associated with all assessed cognitive abilities (*r*s ≥ 0.45, *p*s < 0.001), and global visuospatial cognition (*r* = 0.80 [95% CI: 0.72–0.86], *p* < 0.001). Lifetime JP experience was associated with global visuospatial cognition, even after accounting for other risk and protective factors (β = 0.34 [95% CI: 0.18–0.50], *p* < 0.001). The JP group connected on average 3589 pieces in 49 h. Compared to the counseling group, they improved in JP skill (Cohen’s *d* = 0.38 [95% CI: 0.21–0.54], *p* < 0.001), but not in global visuospatial cognition (Cohen’s *d* = -0.08, [CI: -0.27 to 0.10], *p* = 0.39). The amount of jigsaw puzzling was related to changes in global visuospatial cognition within the JP group, only after accounting for baseline performance (β = 0.33 [95% CI: 0.02–0.63], *p* = 0.03). In sum, our results indicate that jigsaw puzzling strongly engages multiple cognitive abilities and long-term, but not short-term JP experiences could relevantly benefit cognition.

**Trial Registration:** ClinicalTrials.gov **Identifier:** NCT02667314

## Introduction

Preventing cognitive decline in aging such as in mild cognitive impairment and dementia is one of the most relevant medical needs in our aging society (Winblad et al., 2016). Engagement in cognitively, physically, and socially demanding activities is associated with a reduced risk of cognitive decline and dementia in observational studies (Valenzuela and Sachdev, 2006a,b; Wang et al., 2013). Many hundreds of randomized controlled intervention trials have been conducted to investigate the causality of these observational findings. These studies focused mostly on cognitive training programs (Karbach and Verhaeghen, 2014; Lampit et al., 2014), video games (Bediou et al., 2018), and physical exercise such as aerobic exercise and resistance training (Kelly et al., 2014; Young et al., 2015).

However, the cognitive benefits of many other frequently performed leisure activities have not been investigated so far. One example is jigsaw puzzling: alone in Germany, it is estimated that almost 7 million JPs were sold in 2016, resulting in a market of €75 million (Npdgroup Deutschland GmbH, 2016). The jigsaw puzzle (JP) market in Europe and the United States was in total more than €400 million in 2016 (The Npd Group, Inc, 2016; The Npd Group Inc, 2017)^1^, almost twice as much as the worldwide cognitive training market in 2013 (about $220 million; Simons et al., 2016).

Jigsaw puzzling may provide two active ingredients (i.e., effective features) that benefit cognition: first, process-specific cognitive demands of jigsaw puzzling could contribute to an increased brain reserve (Gelfo et al., 2018), and second, regulation of distressing emotions through jigsaw puzzling could prevent chronic stress states that can exert a negative impact on cognitive aging and dementia in the long term (Lupien et al., 2009; Wilson et al., 2011).

Based on a cognitive task analysis, jigsaw puzzling may demand multiple cognitive abilities including visual perception (e.g., recognizing objects, patterns, and orientation of lines), constructional praxis (e.g., integrating visual and motor information to assemble pieces), mental rotation (e.g., mentally rotating piece’s orientation to fit them to other pieces), cognitive speed and visual scanning (e.g., sorting puzzle pieces), cognitive flexibility (e.g., switching attention between different strategies, between different puzzle pieces, and between puzzle shape, image, and color), perceptual reasoning (e.g., integrating different perceptual information to develop strategies and plans how to solve the puzzle), and working and episodic memory (e.g., keeping the association between spatial location and visual patterns/images of puzzle pieces in working memory and long-term memory).

As another potential active ingredient, engaging in jigsaw puzzling could serve to cope with stressors by regulating distressing emotions (Hutchinson et al., 2003). This *emotion-focused coping* (Folkman and Lazarus, 1980) through JPs can be subdivided in two *leisure coping strategies* (Iwasaki and Mannell, 2000): first, jigsaw puzzling could depict a *leisure palliative coping* (Iwasaki and Mannell, 2000) or in other words a “breather” from stress (Lazarus et al., 1980), which may result from its focused attentional demands that enables a psychological time out from stressors. Second, jigsaw puzzling can serve as a *mood enhancement* through fun, flow, and mastery experiences.

Despite the frequent use of jigsaw puzzling as a leisure activity and its potential effects on cognition as a cognitive demanding activity and emotion-focused coping strategy, the role of jigsaw puzzling in cognitive aging has not been specifically investigated in observational and interventional designs so far.

To our knowledge, there is no randomized controlled trial on the effects of jigsaw puzzling and only three observational studies that give hints to the cognitive demands and effects of jigsaw puzzling. In cognitively healthy children, Dykens (2002) found strong associations of jigsaw puzzling performance with a figure copy task and two puzzle-like visuospatial intelligence tests (*r*s = 0.59–0.72). While this study indicates that jigsaw puzzling is cognitively challenging, two studies provide evidence for potential cognitive effects. Levine et al. (2012) found a positive association between the frequency of puzzle play (including jigsaw puzzling) between 2 and 4 years of age and 2-D spatial transformation skills at 4.5 years of age. While many studies demonstrated lifetime cognitive activity as a protective factor for cognitive aging (Vemuri et al., 2014) and dementia (Lee et al., 2018), to our knowledge, only one study included jigsaw puzzling in the composite score of cognitive activity (Friedland et al., 2001). This study did not report specific results on jigsaw puzzling.

Here, we aimed to close the science-practice gap between a lack of research on the cognitive demands and effects of jigsaw puzzling and its frequent use as a leisure activity. First, we evaluated whether and which visuospatial cognitive abilities are tapped by solving JPs. Second, we assessed whether lifetime JP experience is a protective factor for visuospatial cognitive aging in an observational design. As protective factors found in observational designs are not manipulated and hence not necessarily causal (National Research Council and Institute of Medicine, 2009), we investigated the impact of a 30-day JP intervention on visuospatial cognition in a randomized, assessor-blinded, controlled clinical trial. Finally, we aimed at revealing a dose-response relationship between the amount of jigsaw puzzling and visuospatial cognitive improvement within the JP group.

## Materials and Methods

The observational study and randomized, controlled, assessor-blinded superiority trial with two parallel groups was conducted from March 1 to September 12, 2016, at Ulm University, Germany. The study protocol article on the randomized controlled trial has been published^2^ (Fissler et al., 2017), the study was preregistered^3^, and the CONSORT Checklist is provided in **Supplementary Table S1**.

### Procedure

The Ethics Committee of Ulm University approved the study. We described the methods in more detail in the study protocol (Fissler et al., 2017). All participants gave written informed consent prior to participation and received 40€ as financial compensation. We recruited participants via newspaper articles and flyers. Eligibility was assessed in a telephone-based pre-screening (t_1_) and an on-site screening at Ulm University before the pretest assessment (t_2_). Eligible participants completed a 1.5-h pretest. Subsequently, we provided a 15-min cognitive health counseling and disclosed the group allocation. The 30-day intervention period started within 2 weeks after the pretest. During the intervention period, all participants were contacted via telephone three times (t_3_–t_5_). After the intervention, the posttest (t_6_) was scheduled within 2 weeks.

### Eligibility Criteria

Inclusion criteria were a minimum age of 50 years, low JP experience within the past 5 years (<5 completed JPs), interest in solving JPs, commitment of spending 30 h with solving JPs, intact vision and motor function of the upper extremities, and unimpaired cognition [Mini Mental State Examination (MMSE; Folstein et al., 1975) ≥ 24]. Exclusion criteria were self-reported psychiatric, neurologic, or other diseases potentially influencing changes in cognitive performance and current participation in another intervention study.

### Interventions

Participants were randomly allocated to solving JPs plus cognitive health counseling (JP group) or cognitive health counseling only (counseling group).

#### Cognitive Health Counseling

Participants received general information about modifiable lifestyle risk and protective factors for cognitive decline and dementia such as cognitive, physical, and social activity and nutrition (session 1 at t_2_). In the telephone calls during the intervention period, we gave individual feedback regarding potential changes in risk and protective factors based on participants’ individual lifestyle (session 2 at t_3_). As the last behavioral change strategy, we monitored behavior changes by asking participants for changes in the respective lifestyle domains (sessions 3 and 4 at t_4_ and t_5_).

#### Jigsaw Puzzle Intervention

The JP group was instructed to solve JPs at home (30 days within 5 weeks, ≥1 h/day). The first JP was standardized (*Beautiful Prague*, 300 pieces; by Ravensburger Spieleverlag GmbH, RSV), while all subsequent JPs were freely selected by the participants (200–1500 piece JPs). However, we encouraged participants to increase difficulty of the JPs by increasing the number of pieces per JP as long as this did not reduce their pleasure and fun. Recommendations were based on JP performance (time per connected piece) and participants’ reports about perceived difficulty, challenge, pleasure, and fun that were assessed in each of the three telephone interviews (Fissler et al., 2017). To monitor protocol adherence closely, participants received a diary and were asked to fill it out immediately after each session. For each intervention day, participants were supposed to note the respective JP name, duration, and optional comments about their puzzle experience. After each of three cognitive health counseling sessions (t_3-5_), participants had to report their diary entries via telephone.

### Randomization and Blinding

We applied stratified, blocked randomization with two age bands (50–64 years; ≥65 years) and two cognitive status bands (MMSE: 24–27; 28–30). We randomly allocated participants to the two groups in each stratum in blocks of four with a 1:1 allocation ratio. An author uninvolved in data collection (LL) generated the allocation sequence^4^, which was concealed in numbered envelopes for each stratum.

The staff involved in enrollment (OK, PF, and DL), assessment (DL and HA), and on-site cognitive health counseling (OK and PF) were blind to group assignment, which was disclosed to participants after on-site counseling (OK and PF). They were instructed verbally and in a written and signed form not to disclose group assignment to the outcome assessors. However, two participants disclosed group assignment before completion of the primary outcome assessment of the posttest.

### Outcomes

#### Primary and Secondary Outcomes

As primary outcome, a composite score representing global visuospatial cognition was created by averaging eight *z*-standardized domain scores: visual perception (adapted from Benton’s Judgment of Line Orientation Test; Benton et al., 1994), visuospatial processing speed and flexibility (Trail Making Test part A and B; Reitan, 1958; Welsh et al., 1994), visuospatial working memory (Visual Memory Span, Wechsler Memory Scale-Revised, German edition; Härting et al., 2000), constructional praxis and visuospatial episodic memory (Rey Complex Figure Test; Osterrieth, 1944), mental rotation [adapted version of the Mental Rotations Test–Letters (Neuburger et al., 2011) and Mental Rotations Test A (Peters et al., 1995)], and visuospatial reasoning (Block Design, Wechsler Adult Intelligence Scale–III, German edition; Von Aster et al., 2006). As pre-defined in the study protocol (Fissler et al., 2017), ability scores with skewness over |1| were Blom-transformed (Blom, 1958; Ball et al., 2002) in order to minimize ceiling and floor effects and increase reliability (retest reliability without transformation, *r* = 0.88; with transformation, *r* = 0.90).

#### Other Measures

We assessed age, gender, education, and profession in an interview. Adverse events were recorded at the telephone calls (t_3_–t_5_) and the posttest (t_6_).

We asked for modifiable lifestyle risk and protective factors for dementia, among others cardiovascular risk factors, Mediterranean diet (Martínez-González et al., 2012), and the number of recently performed cognitive, physical, and social activity types (Challenging Leisure Activities Questionnaire, based on the CHAMPS Physical Activity Questionnaire for Older Adults; Stewart et al., 2001).

We assessed JP skill as the average time per connected piece for frame and inner parts of a 40 piece JP (*Rothenburg ob der Tauber*, by RSV). Within the JP group, we also assessed the self-reported time for solving the first 300 piece JP in the intervention for an additional analysis (*Beautiful Prague*, by RSV).

Participants retrospectively rated their lifetime experience in solving JPs from 1 (*none*) to 5 (*very high*) and estimated the time spent with solving JPs over the lifetime from 1 (*<50 h*) to 5 (*>350 h*) and the number of connected JP pieces from 1 (*<2000 pieces*) to 5 (*>8000 pieces*). The mean of all three ratings served as measure of lifetime JP experience.

The average of the *z*-standardized time spent with solving JPs and of the number of completed JP pieces during the intervention period served as a measure of the amount of solving JPs.

Participants subjectively estimated the effects of the interventions at the last telephone interview (t_5_) before the posttest (t_6_). They were asked whether they expected that their performance in the visuospatial cognitive tests at the posttest would be positively influenced (*yes* or *no*) and to estimate how their performance would change from pretest to posttest from 1 (*improve markedly*) to 5 (*decline markedly*). Both questions were also asked regarding JP skill. After primary outcome assessment at the posttest, we asked the participants how motivated they were while performing the visuospatial cognitive tests from 1 (*not at all*) to 5 (*very much*).

### Statistical Analysis

G^∗^Power and the R software package were used for statistical analyses (Faul et al., 2007; R Development Core Team, 2015). We calculated the power to detect associations with medium effect size (*r* = 0.30) and intervention effects with small effect size (*f* = 0.10), given a retest correlation of 0.90 for the primary outcome and a two-sided α-error of 0.05. The power to detect intervention effects was approximately 99%. The power to detect associations of the primary outcome with the amount of solving JPs was 60%, and with both JP skill and lifetime JP experience 87%.

Associations were evaluated with linear regression analyses (visuospatial cognition and lifetime JP experience, amount of jigsaw puzzling and visuospatial cognitive change) or Pearson correlations (visuospatial cognition and JP skill).

Intervention effects were assessed with linear mixed effects models with time and group as fixed effects and subject as random intercept in a modified intention-to-treat analysis (all participants with follow-up data). Cohen’s *d* served as effect size measure. Analyses regarding the eight secondary outcomes and the two dosage parameters (JP pieces and time) were adjusted for multiple comparisons using Holm’s method.

## Results

**FIGURE 1:**
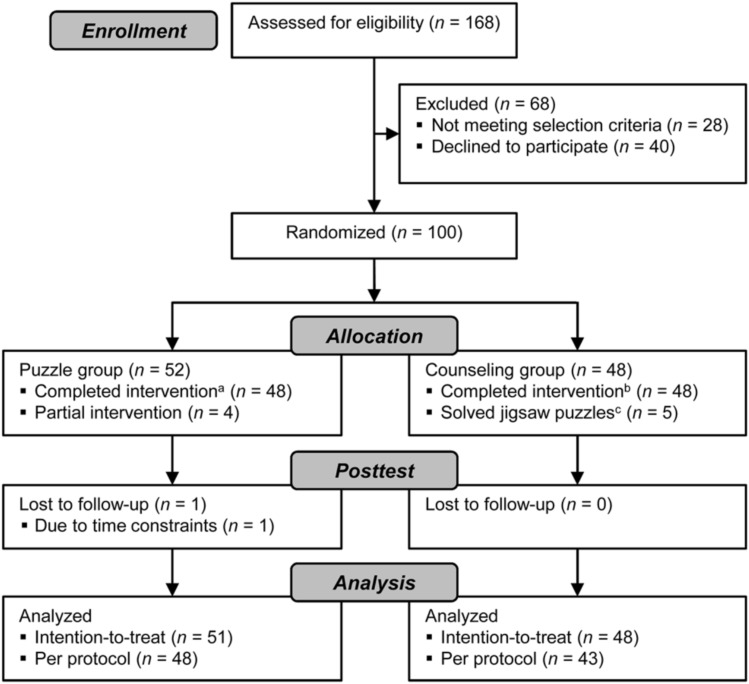
CONSORT flow chart. ^a^Completed at least 24 days with a minimum of 45 min and at least three of four cognitive health counseling sessions, ^b^completed at least three of four cognitive health counseling sessions, and ^c^participants reported solving jigsaw puzzles in the intervention period.

From March 1 to August 3, 2016, we screened 168 interested adults for eligibility, of which 100 participants (64 women, 36 men) were included into the study (see **Figure 1**). Data collection was completed on September 12, 2016. Forty individuals refused study participation after detailed study information, 28 participants did not meet eligibility criteria. Out of 100 included adults, 52 were randomized to the JP group and 48 to the counseling group. One participant was lost to follow-up.

Baseline demographic (age, gender, education) and cognitive characteristics (MMST, global visuospatial cognition) in the two groups were well balanced and differences were below a small effect size (Cohen’s *d* ≤|0.19| ; see **Supplementary Table S2**).

### Association Between Visuospatial Cognition and Jigsaw Puzzle Skill

**FIGURE 2:**
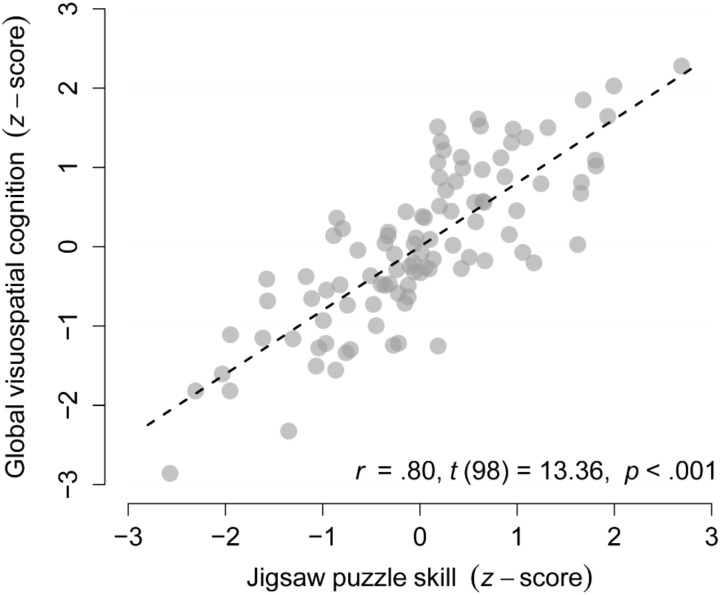
Cognitive demands of solving jigsaw puzzles. Association between jigsaw puzzle skill and global visuospatial cognition in the complete sample (*n* = 100).

Jigsaw puzzle skill, as measured with an untrained 40 piece JP, was highly associated with global visuospatial cognition (*r* = 0.80, *p* < 0.001, see **Figure 2**) and with all eight visuospatial cognitive abilities (*r*s ≥ 0.45, *p*s < 0.001; see **Supplementary Table S3**). Similarly, self-reported time to complete the 300 piece JP was associated with global visuospatial cognition (*r* = 0.70, *p* < 0.001) and with all eight visuospatial cognitive abilities (*r*s ≥ 0.29, *p*s < 0.04; see **Supplementary Results S1** and **Supplementary Table S3**).

### Association Between Global Visuospatial Cognition and Lifetime Jigsaw Puzzle Experience

**FIGURE 3:**
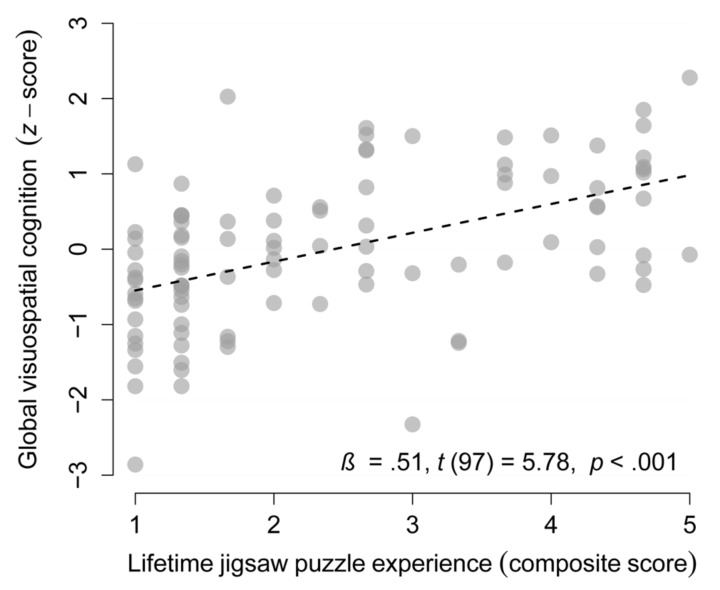
Lifetime jigsaw puzzles experience as a protective factor of cognitive aging. Association between lifetime jigsaw puzzle experience and global visuospatial cognition in the complete sample (*n* = 99, one missing value).

Lifetime JP experience was associated with global visuospatial cognition at baseline (β = 0.51, *p* < 0.001, see **Figure 3**) and with all eight visuospatial cognitive abilities (βs ≥ 0.28, *p*s ≤ 0.008; see **Supplementary Table S4**). Importantly, the associations with global visuospatial cognition, and two secondary outcomes (visuospatial reasoning and cognitive flexibility) remained significant after accounting for the known risk and protective factors age, education, and the number of recently performed social, physical, and cognitive activity types (βs ≥ 0.32, *p*s ≤ 0.004; see **Supplementary Tables S4, S5**).

### Intervention Effects

**FIGURE 4:**
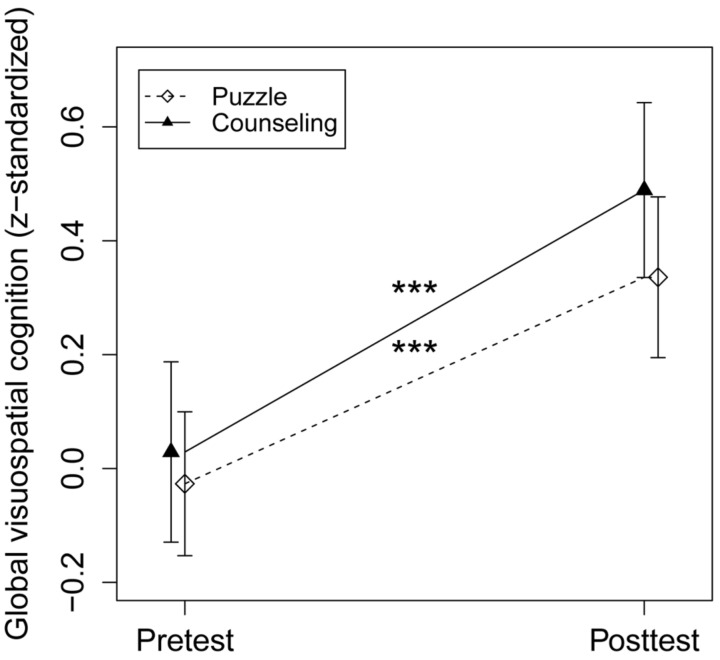
Intervention effect on the primary outcome. The puzzle group showed a pretest–posttest improvement of *d* = 0.38, *p* < 0.001; the counseling group of *d* = 0.46, *p* < 0.001, with an intervention effect size in favor of the counseling group of *d* = 0.08, *p* = 0.39; ^∗∗∗^*p* < 0.001.

Jigsaw puzzle skill improved significantly in the JP group compared to the counseling group (Cohen’s *d* = 0.38 [95% CI: 0.21–0.54], *F* (97) = 20.43, *p*s ≤ 0.001). However, the primary outcome global visuospatial cognition and all secondary outcomes did not show significant group × time interactions (see **Figure 4** and **Table 1**). From pretest to posttest, both groups improved in global visuospatial cognition, mental rotation, processing speed, cognitive flexibility, and episodic memory (Cohen’s *d* ≥ 0.20; *p*s ≤ 0.05). The JP group also improved in reasoning (Cohen’s *d* = 0.20, *p* = 0.02). The supportive per-protocol analysis and pre-defined additional analyses that accounted for confounding variables yielded consistent results.

**Table 1:** Intervention effects on jigsaw puzzle skill and visuospatial cognition.

Measure		Group	Pretest	Posttest	Pre-posttest		Training benefit		
Name	*r*_reliability_	Name	Score	Score	Change^a^	[95% CI]^b^	Cohen’s *d*^b^	[95% CI]^c^	*p*-value^d^
Jigsaw puzzle skill	0.90	Jigsaw puzzle	–0.10	0.33	0.46	[0.35 to 0.57]	0.38	[0.21 to 0.54]	<0.001
		Counseling	0.10	0.19	0.08	[–0.04 to 0.21]			
Global visuospatial cognition	0.90	Jigsaw puzzle	–0.03	0.34	0.38	[0.25 to 0.51]	–0.08	[–0.27 to 0.10]	0.39
		Counseling	0.03	0.49	0.46	[0.32 to 0.60]			
Visual perception	0.72	Jigsaw puzzle	–0.06	–0.20	–0.11	[–0.31 to 0.09]	–0.27	[–0.58 to 0.03]	0.50
		Counseling	0.06	0.23	0.16	[–0.07 to 0.40]			
Constructional praxis	0.42	Jigsaw puzzle	0.16	0.27	0.11	[–0.19 to 0.40]	–0.14	[–0.55 to 0.27]	>0.99
		Counseling	–0.17	0.08	0.25	[–0.04 to 0.54]			
Mental rotation	0.83	Jigsaw puzzle	0.04	0.24	0.23	[0.06 to 0.41]	–0.31	[–0.55 to -0.07]	0.08
		Counseling	–0.05	0.50	0.54	[0.38 to 0.70]			
Processing speed	0.71	Jigsaw puzzle	–0.07	0.17	0.24	[0.05 to 0.43]	–0.01	[–0.30 to 0.28]	>0.99
		Counseling	0.08	0.33	0.25	[0.02 to 0.48]			
Cognitive flexibility	0.74	Jigsaw puzzle	–0.05	0.21	0.26	[0.05 to 0.48]	0.06	[–0.23 to 0.35]	>0.99
		Counseling	0.06	0.26	0.20	[0.005 to 0.40]			
Working memory	0.72	Jigsaw puzzle	–0.13	0.05	0.20	[–0.02 to 0.42]	0.03	[–0.27 to 0.33]	>0.99
		Counseling	0.15	0.32	0.17	[–0.04 to 0.38]			
Episodic memory	0.65	Jigsaw puzzle	0.04	1.01	0.97	[0.72 to 1.21]	0.07	[–0.27 to 0.42]	>0.99
		Counseling	–0.05	0.85	0.90	[0.65 to 1.15]			
Reasoning	0.84	Jigsaw puzzle	–0.08	0.11	0.20	[0.03 to 0.36]	0.12	[–0.11 to 0.35]	>0.99
		Counseling	0.08	0.16	0.08	[–0.09 to 0.24]			


*CI = confidence intervals.*

*^a^positive values indicate beneficial change scores.*

*^b^positive values indicate benefits of the jigsaw puzzle group.*

*^c^confidence intervals are not adjusted for multiple comparisons.*

*^*d*^*p*-values of the group × time interaction of the mixed effects model including 99 participants for each outcome.*

### Dose–Response Relationship

**FIGURE 5:**
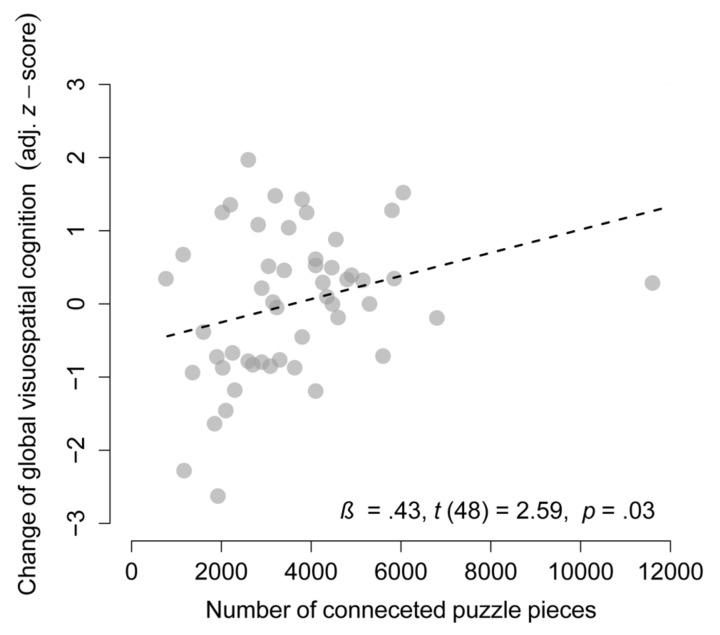
Dose–response analysis. Association between the number of connected puzzle pieces and change in global visuospatial cognition (adjusted for baseline performance) in the puzzle group (*n* = 51, one missing value).

Despite the lack of an effect of the JP intervention, compared to the counseling group, on visuospatial cognition, the dose–response analysis revealed an association of the change in global visuospatial cognition from pretest to posttest with the amount of jigsaw puzzling and with the number of connected JP pieces within the JP intervention, only after accounting for baseline performance to adjust for the regression-to-the-mean effect [β = 0.33*, p* = 0.03, and β = 0.43*, p_(Holmcorrected)_* = 0.03, respectively, see **Figure 5** and **Supplementary Table S6**]. A *post hoc* analysis that removed one influential data point of a participant with a very high amount of jigsaw puzzling revealed a robust dose–response relationship with an even increased effect size for the primary outcome (β = 0.39, *p* = 0.01).

Based on the linear model parameters of the dose–response analysis and the observed pretest–posttest gains in the counseling group, we estimate that individuals would need to connect 9108 JP pieces to induce gains in global visuospatial cognition of medium size (Cohen’s *d* = 0.5).

### Assessment of Opportunity Costs and Placebo Effects

The counseling group increased the number of performed cognitive, physical, and social leisure activity types (excluding JP solving) by 1.4, which is a marginally significant increase relative to the JP group (*p* = 0.07). This indicates that increased engagement in one activity type – such as solving JPs – goes along with a reduced potential of gain from other activity types (opportunity costs). Thus, the use of a counseling control group efficiently accounted for such opportunity costs.

Placebo effects may be induced through differences in expectations, test motivation, and randomization-related disappointment. Participants’ expectations about improvements in cognitive test performance were higher in the JP group than in the control group (76 vs. 33%, *p* < 0.001). Despite this group difference, expectations that performance will improve in the visuospatial tests from pre-to-posttest were positive in the control group (*p* < 0.001, Cohen’s *d* = 0.62). Furthermore, expectations were not related to the amount of solving JPs within the JP group (see **Supplementary Results S2** for more details). Most importantly, expectations were not related to actual cognitive changes and statistically accounting for expectations did not alter group effects and dose–response relationships (see **Supplementary Results S2**).

Test motivation at posttest did not differ significantly between the groups and was not related to the amount of solving JPs within the JP group (see **Supplementary Results S3**). Taken together, the counseling group controlled for the potential gains from alternative activity types, test motivation, and practice effects, but only partly for expectations of cognitive gains. Statistically accounting for expectations did not change our findings and expectations were not related to objective cognitive changes. Therefore, we efficiently took opportunity costs into account and potential placebo effects seemed not to have affected our results.

### Adverse Events

The total number of adverse events did not differ significantly between the groups. The number of adverse events probably due to the intervention were all temporarily, but were significantly higher in the JP group, compared to the control group (*n* = 11 vs. *n* = 0, *p* = 0.005). These events included back and shoulder pain (*n* = 4), loss of motivation in puzzling (*n* = 3), “craving” to solve JPs (*n* = 3), and uncommon headache (*n* = 1; **Supplementary Results S4** and **Supplementary Table S7**).

## Discussion

### Principal Findings

We found that JP skill was highly associated with global visuospatial cognition and all measured visuospatial cognitive abilities indicating that solving JPs strongly taps multiple visuospatial cognitive processes including perception, constructional praxis, mental rotation, speed, flexibility, working memory, reasoning, and episodic memory. This result is in line with a study with cognitively healthy children that found strong correlations (*r*s > 0.5) of JP performance with a figure copy task and two visuospatial intelligence tests that were structurally similar to jigsaw puzzling (Dykens, 2002).

We revealed that self-reported lifetime JP experience was associated with visuospatial cognition in healthy adults above 50 years of age, even after accounting for known predictive factors for cognitive aging. However, this association may be due to an effect of jigsaw puzzling on cognition, vice versa, or due to non-measured confounding variables (e.g., people who solve more JPs may play more games in general). Our results are in line with two observational studies in children (Levine et al., 2012) and older adults (Friedland et al., 2001) that found an association of cognitive outcomes with activity composite scores that included jigsaw puzzling.

Thirty days of solving JPs improved JP skill in an untrained JP, but did not improve global visuospatial cognition, in comparison to the counseling group, in a clinically relevant way. Both groups improved in global visuospatial cognitive performance, which may reflect practice effects through repeated testing, true cognitive benefits in both groups, or a mixture of both. Cognitive benefits have been regularly found in both experimental and active control conditions of intervention studies without significant differences between groups. These results have often been interpreted as true intervention effects in both groups (e.g., Erickson et al., 2011; Barnes et al., 2013; Ballesteros et al., 2017). However, practice effects should not be underestimated, especially with regard to episodic memory tests as subjects know about the delayed recall trial at the second testing (Woods et al., 2006).

Finally, we found a marginally significant dose–response association between the amount of jigsaw puzzling and improvement in visuospatial cognition within the puzzle group that turned significant after accounting for baseline cognition. This association supports the interpretation that cognitive test improvements in the JP group were at least partly due to true cognitive gains. Assuming that our estimated model parameters of this association are true, the association was fully due to an effect of jigsaw puzzling on cognition, we expect that at least ∼9100 JP pieces need to be connected for clinically relevant cognitive gains, in contrast to the control group (Cohen’s *d* = 0.5).

### Implications

Jigsaw puzzling recruits multiple visuospatial cognitive abilities and is a potential protective factor for cognitive aging. Engaging in low amounts of jigsaw puzzling over a 30-day period (approximately 3600 connected pieces) does not improve cognition in a clinically relevant way compared to engaging in other potentially beneficial activities. Our results strengthen the evidence that cognitively demanding activities benefit cognition over the long term, but are no “quick-fix” solution to improve cognition (Gathercole, 2014). Our findings are especially important as solving JPs is a frequently performed and easily applicable leisure activity: it can be executed alone or in groups at almost all places without the need of technical devices, language capabilities, or prior knowledge. Given that solving JPs has no known harms, we think that long-term, but not short-term jigsaw puzzling can be considered for recommendations regarding healthy cognitive aging as one component within an intrinsically motivated, physically, socially and cognitively active lifestyle.

### Strengths, Limitations, and Future Perspectives

This randomized, controlled, assessor-blinded clinical trial conforms to best-practice standards of cognitive intervention trials (Simons et al., 2016). However, there is no consensus regarding a gold-standard control group (Boot et al., 2013). We used a counseling group to control for the potential cognitive benefits through other activity types when solving JPs is chosen (opportunity costs). In addition, we aimed to control for placebo effects induced through group differences in test motivation and expectations about cognitive benefits.

We efficiently accounted for potential opportunity costs, as we did not use a control group of a theoretically inefficient alternative activity type and the counseling group engaged in 1.4 more challenging leisure activity types during the intervention period than the JP group (excluding solving JPs). However, we cannot exclude that solving JPs may be superior in inducing cognitive benefits compared to activities, with no expected influence on cognition. To evaluate this, future studies could include a second control condition using a theoretically inefficient activity type such as watching television (Lindstrom et al., 2005).

We implemented three strategies to prevent placebo effects due to higher expectations and test motivation in the JP group, as well as due to disappointment in the control group: first, cognitive health counseling for the control group; second, verbally lowering expectations for jigsaw puzzling effects and increasing expectations for effects through other behavioral changes; and third, high amount of study staff contact in both groups. However, we still found higher expectation regarding benefits in visuospatial cognitive task performance in the JP group than in the counseling group. Importantly, the counseling group had significantly positive expectations, statistically accounting for expectations, did not alter our results, the amount of solving JPs and the cognitive improvements were not related to expectations, and finally, test motivation did not differ between groups at posttest. In behavioral interventions, participants are *per se* aware of their behaviors and eliminating group differences in differentially effective interventions may need stronger verbal manipulation of expectations or control activities which induce a mismatch of effects and expectations. To account for potential effects of randomization-related disappointment on cognitive change, it should be measured after group assignment in future trials.

As the choice of tasks is critical for assessing transfer to broad cognitive abilities, we used a composite measure of multiple cognitive tests that are structurally dissimilar to jigsaw puzzling. This should prevent that improvements in task-specific skills transferred to gains in cognitive tests. While such composite scores are still the gold standard in measuring broad cognitive gains (Ngandu et al., 2015), future studies may additionally use latent difference score models for a secondary analysis strategy (McArdle, 2009; Schmiedek et al., 2014).

The jigsaw puzzling duration was relatively high with an average of 49 h within 30 days, in comparison to other intervention studies (e.g., action video game studies had an average training duration of 23 h; Bediou et al., 2018). However, the difference between participants’ amount of jigsaw puzzling through the intervention is still small when compared with the differences between participants in the observational study that investigated the experience across the whole lifetime. Based on the results of the dose–response relationship, higher amounts of jigsaw puzzling (>9100 connected pieces) might have the potential to induce relevant cognitive benefits. Therefore, future studies should manipulate the amount of jigsaw puzzling over longer periods (1–2 years) to shed light on the causality of the experience-cognition association (cf., Ngandu et al., 2015).

Finally, future studies should investigate the underlying mechanisms of the association between long-term jigsaw puzzling and cognition. First, process-specific cognitive demands of jigsaw puzzling may induce changes at a cellular level (synaptogenesis, neurogenesis, gliogenesis, and angiogenesis) and molecular level (changes in neurotransmitters and neurotrophins; Gelfo et al., 2018) resulting in a more efficient neuronal network (McDonough et al., 2015) that better copes with brain pathological or age-related changes (Stern, 2009). Second, the potential of jigsaw puzzling to regulate distressing emotions as a *breather* (Lazarus et al., 1980) and a *mood enhancement* (Iwasaki and Mannell, 2000) may reduce the negative impact of chronic stress on cognition (Lupien et al., 2009; Wilson et al., 2011), e.g., by regulating cortisol levels. Future studies need to investigate these potential mechanisms of action by assessing physiological parameters such as task-related brain activity (McDonough et al., 2015) or resting-state connectivity (Franzmeier et al., 2017), blood-based or hair-based parameters such as brain-derived neurotrophic factor (Harb et al., 2017) or cortisol levels (Koenig et al., 2018). Finally, cognitively demanding leisure activities such as jigsaw puzzling may exert their effects not only by increasing brain reserve but might have the potential to affect brain pathology such as Alzheimer’s disease (Landau et al., 2012). Therefore, associations between lifetime JP experience (next to other leisure activities) and markers of Alzheimer’s disease should be investigated.

## Conclusion

Our findings indicate that jigsaw puzzling recruits multiple visuospatial cognitive abilities and is a – not necessarily causal – protective factor for visuospatial cognitive aging. In addition, we found no evidence that low amounts of jigsaw puzzling over a 30-day period (approximately 3600 connected JP pieces) induce clinically relevant cognitive benefits, compared to engaging in other potentially beneficial activities.

## Data Availability

The raw data supporting the conclusions of this manuscript will be made available by the authors, without undue reservation, to any qualified researcher.

## Author Contributions

PF with supervision of I-TK acquired the financial support for the project and conceptualized the research aims. PF and OK managed and coordinated the research activity planning and execution. PF, LL, CvA, OK, and DL designed the cognitive health counseling and the JP intervention. PF, OK, LL, CvA, and I-TK conceptualized the design and methodology. PF, OK, DL, and LL wrote the initial draft of the manuscript. PF, OK, and DL conducted the data analyses. OK and PF registered the clinical trial. LL conducted the randomization procedure. PF, OK, and DL coordinated availability of study materials. DL coordinated the outcome assessment. PF and OK created the figures of the manuscript, DL, PF, and OK conducted the investigational process (recruitment, pre-screening and screening, cognitive health counseling, outcome assessment, and communication of participants’ results). PF, OK, DL, LL, CvA, and I-TK interpreted the data and reviewed and edited the first draft critically for important intellectual content.

## Conflict of Interest Statement

PF, OK, LL, and DL were employed within the PACE project that is funded by RSV. RSV may gain or lose financially from the publication of this and following manuscripts about the PACE project. The authors declare that they were not influenced in any way by RSV with regard to study design, collection, management, analysis, and interpretation of data, writing of the manuscript, and the decision to submit the report for publication. Apart from providing the JPs, RSV had no role in conducting the study. CvA received honoraria from serving on the scientific advisory board of Nutricia GmbH (2014) and Hongkong University Research council (2014) and has received funding for travel and speaker honoraria from Nutricia GmbH (2014–2015), Novartis Pharma GmbH (2011), Lilly Deutschland GmbH (2013–2017), Desitin Arzneimittel GmbH (2014), Biogen (2016–2017), Roche (2017), and Dr. Willmar Schwabe GmbH & Co. KG (2014–2015) and has received research support from Roche Diagnostics GmbH (2013–2015), Biologische Heilmittel Heel GmbH (2012), and ViaMed GmbH (2011–2014). The authors declare that the study was not influenced in any way by these companies. The remaining author declares that the research was conducted in the absence of any commercial or financial relationships that could be construed as a potential conflict of interest.

## Footnotes

1 NPD data captures roughly 80% of total market penetration. It does not capture all specialty channel data and other accounts. Copyright 2017. The NPD Group, Inc. All Rights Reserved.

2 http://rdcu.be/vEhX

3 https://clinicaltrials.gov/ct2/show/NCT02667314

4 https://www.random.org/
