# Supplementary material for: Jigsaw Puzzling Taps Multiple Cognitive Abilities and Is a Potential Protective Factor for Cognitive Aging
Source: Front Aging Neurosci. 2018 Oct 1;10:299. doi: 10.3389/fnagi.2018.00299 (PMC6174231; doi:10.3389/fnagi.2018.00299)
Supplement: Supplementary file 1 [file Table_1.docx]

Supplementary Material

Jigsaw puzzling taps multiple cognitive abilities and is a potential protective factor for cognitive aging

Patrick Fissler, Olivia Caroline Küster, Daria Laptinskaya, Laura Sophia Loy, Christine A. F. von Arnim, Iris-Tatjana Kolassa

*** Correspondence:** Patrick Fissler: [patrick.fissler@uni-ulm.de](mailto:patrick.fissler@uni-ulm.de)

# Supplementary results

## Results S1. Link between jigsaw puzzle skill and global visuospatial cognition after accounting for covariates

The associations between jigsaw puzzle skill and global visuospatial cognition remained similar after accounting for lifetime jigsaw puzzle experience (40 piece puzzle: *r* = .72, *p* < .001; 300 piece puzzle: *r* = .63, *p* < .001).

## Results S2. Group differences of participant’s expectations regarding cognitive change

In comparison to the counseling group, the JP group expected more often that their performance in the visuospatial cognitive tests was positively affected (76% vs. 33%, respectively; *p* < .001). Similar results were observed for expected gains in JP performance (96% vs. 39%, respectively; *p* < .001). Additionally, the JP group had higher expectations regarding positive performance changes from pre- to posttest in the visuospatial cognitive tests (Cohen’s *d* = 1.3; *p* < .001) and in solving JPs (Cohen’s *d* = 2.0; *p* < .001), in comparison to the counseling group. Despite group differences, also the counseling group expected that their performance in the visuospatial tests will improve in the posttest (*p* < 0.001, Cohen’s *d* = 0.62). The amount of jigsaw puzzling within the JP group was not associated with expectations regarding cognitive changes (*p*s ≥ .76) or changes in JP skill (*p*s ≥ .52). Expectations regarding cognitive changes were not associated with actual cognitive changes (β < 0.0, *p*s ≥ .14), but expectations regarding changes in JP skill were related to actual changes in JP skill (*p*s ≤ .03). Importantly, the effect of the JP group on JP skill as well as the dose-response associations between visuospatial cognitive improvement and the amount of solving JPs were not altered after accounting for respective expectations.

## Results S3. Group differences in test motivation at posttest

Motivation during the neuropsychological tests at the posttest did not differ between the JP group (*M* = 4.2 out of 5; *SD* = 0.6) and the counseling group (*M* = 4.5 out of 5; *SD* = 0.7; Cohen’s *d* = -0.38, *p* = .09) and was not correlated with the amount of jigsaw puzzling (*r* = .09, *p* = .56) or with pre-posttest changes in global visuospatial cognition and JP skill (*p*s ≥ .72).

## Results S4. Group differences in adverse events

The total number of adverse events did not differ between the JP group (*n* = 49) and the counseling group (*n* = 35, *p* = .98). However, there were more adverse events due to the intervention within the JP group (*n* = 11) than within the counseling group (*n* = 0, *p* = .005). All adverse events probably due to the JP intervention were temporarily, including uncomfortable craving for solving JPs (*n* = 3), back and shoulder pain (*n* = 4), frustration or loss of motivation (*n* = 3), and uncommon headache (*n* = 1, see Table S5).

# Supplementary tables

| **Table S1.** CONSORT 2010 checklist of information to include when reporting a randomised trial* | | | |
| --- | --- | --- | --- |
| Section/Topic | Item No | Checklist item | Reported on page No |
| Title and abstract | | | |
|  | 1a | Identification as a randomised trial in the title | Not possible |
|  | 1b | Structured summary of trial design, methods, results, and conclusions | Not possible |
| Introduction | | | |
| Background and objectives | 2a | Scientific background and explanation of rationale | p. 4 |
|  | 2b | Specific objectives or hypotheses | p. 4-5 |
| Methods | | | |
| Trial design | 3a | Description of trial design (such as parallel, factorial) including allocation ratio | p. 5 |
|  | 3b | Important changes to methods after trial commencement (such as eligibility criteria), with reasons | No changes |
| Participants | 4a | Eligibility criteria for participants | p. 5 |
|  | 4b | Settings and locations where the data were collected | p. 5 |
| Interventions | 5 | The interventions for each group with sufficient details to allow replication, including how and when they were actually administered | p. 5-6 and study protocol article |
| Outcomes | 6a | Completely defined pre-specified primary and secondary outcome measures, including how and when they were assessed | p. 7 and ClinicalTrials.gov Identifier: NCT02667314 |
|  | 6b | Any changes to trial outcomes after the trial commenced, with reasons | No changes for the reported part of the study |
| Sample size | 7a | How sample size was determined | p. 8 |
|  | 7b | When applicable, explanation of any interim analyses and stopping guidelines | No interim analysis |
| Randomisation: |  |  |  |
| Sequence generation | 8a | Method used to generate the random allocation sequence | p. 6-7 |
|  | 8b | Type of randomisation; details of any restriction (such as blocking and block size) | p. 6-7 |
| Allocation concealment mechanism | 9 | Mechanism used to implement the random allocation sequence (such as sequentially numbered containers), describing any steps taken to conceal the sequence until interventions were assigned | p. 6-7 |
| Implementation | 10 | Who generated the random allocation sequence, who enrolled participants, and who assigned participants to interventions | p. 6-7 |
| Blinding | 11a | If done, who was blinded after assignment to interventions (for example, participants, care providers, those assessing outcomes) and how | p. 6-7 |
|  | 11b | If relevant, description of the similarity of interventions | Not relevant (component control design) |
| Statistical methods | 12a | Statistical methods used to compare groups for primary and secondary outcomes | p. 9 |
|  | 12b | Methods for additional analyses, such as subgroup analyses and adjusted analyses | p. 9 |
| Results | | | |
| Participant flow (a diagram is strongly recommended) | 13a | For each group, the numbers of participants who were randomly assigned, received intended treatment, and were analysed for the primary outcome | p. 9 & Figure 1 |
|  | 13b | For each group, losses and exclusions after randomisation, together with reasons | p. 9 & Figure 1 |
| Recruitment | 14a | Dates defining the periods of recruitment and follow-up | p. 9 |
|  | 14b | Why the trial ended or was stopped | Was not stopped |
| Baseline data | 15 | A table showing baseline demographic and clinical characteristics for each group | p. 9 & Supplementary Material, Table S2 |
| Numbers analysed | 16 | For each group, number of participants (denominator) included in each analysis and whether the analysis was by original assigned groups | Figure 1 and Table 1 |
| Outcomes and estimation | 17a | For each primary and secondary outcome, results for each group, and the estimated effect size and its precision (such as 95% confidence interval) | Table 1 and p. 10 |
|  | 17b | For binary outcomes, presentation of both absolute and relative effect sizes is recommended | No binary outcomes |
| Ancillary analyses | 18 | Results of any other analyses performed, including subgroup analyses and adjusted analyses, distinguishing pre-specified from exploratory | p. 9-12 and Supplementary Material |
| Harms | 19 | All important harms or unintended effects in each group | p. 12 and Supplementary Material |
| Discussion | | | |
| Limitations | 20 | Trial limitations, addressing sources of potential bias, imprecision, and, if relevant, multiplicity of analyses | p. 13-14 |
| Generalisability | 21 | Generalisability (external validity, applicability) of the trial findings | p. 13 |
| Interpretation | 22 | Interpretation consistent with results, balancing benefits and harms, and considering other relevant evidence | p. 13 |
| Other information | | |  |
| Registration | 23 | Registration number and name of trial registry | p. 2 |
| Protocol | 24 | Where the full trial protocol can be accessed, if available | <http://rdcu.be/vEhX> |
| Funding | 25 | Sources of funding and other support (such as supply of drugs), role of funders | p. 16 |

| **Table S2.** Baseline characteristics of the jigsaw puzzle and counseling group | | | |
| --- | --- | --- | --- |
| **Measure** | **Jigsaw puzzle group (*n* = 52)** | **Counseling group (*n* = 48)** | **Cohen’s *d*** |
| Demographic data |  |  |  |
| Age, mean (*SD*), y | 62.7 (8.4) | 64.0 (7.8) | -0.15 |
| Female, *n* (%) | 36 (69%) | 28 (58%) |  |
| Education, years mean (*SD*), y | 14.2 (2.8) | 13.8 (2.7) | 0.14 |
| Cognitive data |  |  |  |
| MMST, mean (*SD*), points | 28.6 (1.4) | 28.9 (1.1) | -0.19 |
| Global visuospatial cognition | -0.03 (0.9) | 0.03 (1.1) | -0.06 |
| MMST = Mini Mental State Examination | | | |

| **Table S3.** Association between jigsaw puzzle skill and cognition | | |
| --- | --- | --- |
| **Measure** | **Pearson's *r*** | |
|  | **40 piece puzzle (*n* = 100)** | **300 piece puzzle (*n* = 51)** |
| Global visuospatial cognition | .80^a^ | .70^a^ |
| Visual perception | .45^a^ | .35^c^ |
| Constructional praxis | .47^a^ | .37^c^ |
| Mental rotation | .60^a^ | .46^b^ |
| Processing speed | .66^a^ | .53^a^ |
| Cognitive flexibility | .65^a^ | .59^a^ |
| Working memory | .46^a^ | .29^c^ |
| Episodic memory | .44^a^ | .44^b^ |
| Reasoning | .72^a^ | .59^a^ |
| ^a^ *p*-value < .001 | | |
| ^b^ *p*-value < .01 | | |
| ^c^ *p*-value < .05 | | |

| **Table S4.** Association between lifetime jigsaw puzzle experience and cognition | | |
| --- | --- | --- |
| **Measure** | ***β_unadjusted model_^a^*** | ***β_adjusted model_^b^*** |
| Global visuospatial cognition | .51^d^ | .34^d^ |
| Visual perception | .29^e^ | .22^g^ |
| Constructional praxis | .28^e^ | .21^g^ |
| Mental rotation | .32^e^ | .14 |
| Processing speed | .35^e^ | .21^g^ |
| Cognitive flexibility | .47^d^ | .32^e^ |
| Working memory | .33^e^ | .19 |
| Episodic memory | .30^e^ | .24^g^ |
| Reasoning | .46^d^ | .34^e^ |
| ^a^ association without accounting for known risk and protective factors | | |
| ^b^ association accounting for age, education, and recently performed social, physical and cognitive activity types | | |
| ^c^ association accounting for jigsaw puzzle skill, age, education, and lifestyle | | |
| ^d^ *p*-value < .001 | | |
| ^e^ *p*-value < .01 | | |
| ^g^ *p*-value < .05 without adjusting for multiple comparisons of the secondary outcomes | | |

| **Table S5.** Global visuospatial cognition predicted by lifetime jigsaw puzzle experience after accounting for other predictive factors | | | | |
| --- | --- | --- | --- | --- |
| **Model and variable** | **Global visuospatial cognition** | | | |
|  | ***ΔR^2^*** | ***B*** | ***β*** | ***p-*value** |
| Model 1 | .41 |  |  | < .001 |
| Age |  | -0.06 | -.53 | < .001 |
| Education |  | 0.06 | .16 | .06 |
| Activity types |  | 0.78 | .13 | .12 |
| Model 2 | .09 |  |  | < .001 |
| Age |  | -0.05 | -.38 | < .001 |
| Education |  | 0.06 | .16 | .04 |
| Activity types |  | 1.55 | .16 | .04 |
| Jigsaw puzzle experience |  | 0.26 | .34 | < .001 |
| Activity types = number of recently performed social, physical and cognitive activity types | | | | |

| **Table S6.** Association between the amount of puzzling and cognitive change | | | | |
| --- | --- | --- | --- | --- |
| **Pre-posttest change score** | **Amount of jigsaw puzzling^a^** | | **Puzzle pieces** | **Puzzle time** |
|  | ***β_unadjusted model_^b^*** | ***β_adjusted model_^c^*** | ***β_adjusted model_^c^*** | ***β_adjusted model_^c^*** |
| Global visuospatial cognition | .27^e^ | .33^d^ | .43^d^ | .23 |
| Visual perception | .17 | .22 | .19 | .21 |
| Constructional praxis | .12 | .26^f^ | .33^e,f^ | .16 |
| Mental rotation | .17 | .22 | .29^f^ | .12 |
| Processing speed | -.12 | .14 | .19 | .08 |
| Cognitive flexibility | -.08 | .06 | .19 | .05 |
| Working memory | .30^f^ | .34^f^ | .42^d^ | .21 |
| Episodic memory | .26 | .31^f^ | .35^f^ | .23 |
| Reasoning | -.04 | .09 | .15 | .03 |
| ^a^ composite score of the number of connected jigsaw puzzle pieces and the jigsaw puzzle time | | | | |
| ^b^ without accounting for baseline scores | | | | |
| ^c^ accounting for baseline scores to reduce the regression-to-the-mean effect | | | | |
| ^d^ *p*-Value < 0.05 | | | | |
| ^e^ *p*-Value < 0.10 | | | | |
| ^f^ *p*-Value < 0.05 without adjusting for multiple comparisons of the eight secondary outcomes and the two dosage parameters | | | | |

| **Table S7.** Adverse events | | | |
| --- | --- | --- | --- |
| **Measure** | **Jigsaw puzzle group (*n*= 52)** | **Counseling group (*n*= 48)** | ***p*-value^a^** |
| AEs, mean (*SD*), *n* | 0.94 (1.5) | 0.73 (0.9) | .98 |
| AEs due to intervention^b^, mean (*SD*), *n* | 0.21 (0.5) | 0.00 (0.0) | .005 |
| Back or shoulder pain, mean (*SD*), *n* | 0.08 (0.3) | 0.00 (0.0) | .05 |
| Craving to solve jigsaw puzzles, mean (*SD*), *n* | 0.06 (0.2) | 0.00 (0.0) | .10 |
| Loss of motivation, mean (*SD*), *n* | 0.06 (0.2) | 0.00 (0.0) | .10 |
| Uncommon headache, mean (*SD*), *n* | 0.02 (0.1) | 0.00 (0.0) | .35 |
| AE = adverse event | | | |
| ^a^ Mann-Whitney *U* test without adjusting for multiple comparisons | | | |
| ^b^ all adverse events were temporarily | | | |
